# The Metacaspase *TaMCA-Id* Negatively Regulates Salt-Induced Programmed Cell Death and Functionally Links With Autophagy in Wheat

**DOI:** 10.3389/fpls.2022.904933

**Published:** 2022-06-23

**Authors:** Jie-yu Yue, Ying-jie Wang, Jin-lan Jiao, Wen-wen Wang, Hua-zhong Wang

**Affiliations:** Tianjin Key Laboratory of Animal and Plant Resistance, Tianjin Normal University, Tianjin, China

**Keywords:** autophagy, metacaspase, NaCl stress, PCD, *TaMCA1*, wheat seedling

## Abstract

Metacaspases (MCAs), a family of caspase-like proteins, are important regulators of programmed cell death (PCD) in plant defense response. Autophagy is an important regulator of PCD. This study explored the underlying mechanism of the interaction among PCD, MCAs, and autophagy and their impact on wheat response to salt stress. In this study, the wheat salt-responsive gene *TaMCA-Id* was identified. The open reading frame (ORF) of *TaMCA-Id* was 1,071 bp, coding 356 amino acids. The predicted molecular weight and isoelectric point were 38,337.03 Da and 8.45, respectively. *TaMCA-Id* had classic characteristics of type I MCAs domains, a typical N-terminal pro-domain rich in proline. *TaMCA-Id* was mainly localized in the chloroplast and exhibited nucleocytoplasmictrafficking under NaCl treatment. Increased expression of *TaMCA-Id* in wheat seedling roots and leaves was triggered by 150 mM NaCl treatment. Silencing of *TaMCA-Id* enhanced sensitivity of wheat seedlings to NaCl stress. Under NaCl stress, *TaMCA-Id*-silenced seedlings exhibited a reduction in activities of superoxide dismutase (SOD), peroxidase (POD), and catalase (CAT), higher accumulation of H_2_O_2_ and ${\text{O}}_{2}^{.-}$, more serious injury to photosystem II (PSII), increase in PCD level, and autophagy activity in leaves of wheat seedlings. These results indicated that *TaMCA-Id* functioned in PCD through interacting with autophagy under NaCl stress, which could be used to improve the salt tolerance of crop plants.

## Introduction

As major environmental stress, salt stress impaired plant growth, especially at the seedling stage, limited crop yield, and became a danger to food security (Moukhtari et al., 2020). Salt stress harms plant growth in multiple ways, such as ion toxicity, alteration of biochemical, physiological, and metabolic processes, causing osmotic stress, water stress, nutritional disorders, genotoxicity, membrane disorganization, and inhibition of cell division (Wang et al., 2011; Yang et al., 2018; Yu et al., 2020). The plant salt-stress responses were associated with excess accumulation of reactive oxygen species (ROS) that contribute to the interrelationship among the salt-sensitive syndromes (He et al., 2020). ROS have dual functions, i.e., they can produce oxidative damage and initiate cell signaling (Mittler, 2017). Superoxide anion (${\text{O}}_{2}^{.-}$) and hydrogen peroxide (H_2_O_2_) are the two main ROS (Beltrán González et al., 2020). Plants have developed complicated defense mechanisms against salt stresses, containing osmotic adjustment, ion homeostasis, and ROS scavenging (Yu et al., 2020). It has reported that both programmed cell death (PCD) and autophagy regulate plant tolerance to stress conditions, which are associated with ROS accumulation (Zhou et al., 2018a; Yue et al., 2021a).

The PCD is an orderly cell suicide to eliminate unwanted or damaged cells during plant development and against multiple stresses, which is regulated by multifactors (Locato and De Gara, 2018; Yue et al., 2021a; Zhou et al., 2021). Caspases, a family of cysteine-dependent aspartate-directed proteases, are important regulators of PCD (Ojha et al., 2015). Many caspases have an active site and need aspartate residues in the protein substrates for cleavage (Julien and Wells, 2017). Although the caspases have not yet been found in plants, it has been reported that caspase-like enzymatic activity functions in many cell death pathways in plants (Luan et al., 2021). Nevertheless, metacaspase (MCA), a class of cysteine proteases present in plants, is structurally related to caspase and has caspase-like catalytic domain containing 20 kDa (p20) and 10 kDa (p10) subunits that are crucial for mediating PCD (Fagundes et al., 2015; Bansal et al., 2020). MCA includes type I and type II, according to its protein structure (Yao et al., 2020). Type I MCAs have a typical N-terminal pro-domain rich in proline, and Type II MCAs contain a linker region of 160–180 amino acids between the p20 and p10 subunits, but lack the typical N-terminal pro-domain (Fagundes et al., 2015). In the last decade, the biological functions of MCAs have been well-characterized. There were nine *MCA* genes in *Arabidopsis* (*AtMCA1* to *AtMCA9*), which were categorized into Type I and Type II. Type I contained *AtMCA1* to *AtMCA3*, and Type II contained *AtMCA4* to *AtMCA9* (Coll et al., 2010). Besides, the MCs family has been systematically characterized, and the structural and functional features of MCAs were elucidated in rice (Wang and Zhang, 2014), *Viridiplantae* (Fagundes et al., 2015), tomato (Liu et al., 2016b), cucumber (Zhou et al., 2018b), *Rosaceae* (Cao et al., 2019), *Gossypium* species (Fan et al., 2019), and maize (Luan et al., 2021).

Growing evidence has confirmed that MCAs functioned in signaling, stress acclimation, and PCD pathways in plants, which acted positively or negatively to mediate PCD in plant response to stress. For example, Liu et al. (2016a) indicated that many *HbMCAs* involved in the regulation of the drought, cold, and salt stress caused cell death. Hao et al. (2016) found that silencing *TaMCA1* promoted disease resistance of wheat seedlings to *Pst* using virus-induced gene silencing (VIGS). Yao et al. (2020) showed that *AhMCA1* positively regulated Al-induced PCD in root tips of peanut plants through inhibiting the activities of antioxidant enzymes and increasing ROS accumulation. Fernandez et al. (2021) found that both *MoMCA1* and *MoMCA2* played a vital role in oxidative stress-induced apoptosis and had an important role in sporulation and subsequently pathogenicity during rice blast disease progression.

Autophagy, a catabolic intracellular nutrient-scavenging pathway, has a vital role in regulating plant responses to multiple stresses (Wang et al., 2021). It has been verified that there is an intrinsic relation between autophagy and PCD in plant stress response (Yue et al., 2021a; Zhou et al., 2021). When autophagy is triggered by stress, intracellular remnants are sequestered into the double-membrane autophagosomes and subsequently delivered to the vacuoles for degradation in plants (Üstün et al., 2017). Both autophagosome formation and autophagosome-vacuole fusion required induction of a set of autophagy-related genes (ATGs) and activation of specific proteases, such as MCAs (Coll et al., 2014). Minina et al. (2013) indicated that activation of autophagy requires type II MCA mcII-Pa, and silencing of *mcaII-Pa* resulted in a similar result with the symptoms observed in autophagy-inhibited plants of *Norway spruce*, implicating that *mcII-Pa* was an upstream regulator of autophagy in the embryogenesis. Berenguer et al. (2020) demonstrated that both MCA and autophagy were activated and eventually dictated cell death during microspore embryogenesis under heat stress. However, the interplay among MCAs, PCD, and autophagy in wheat seedlings' response to salt stress has not been clarified. Therefore, we focused on the function of *TaMCA-Id-4D* in response of wheat seedlings to salt stress, which was cloned from wheat seedlings. Barley stripe mosaic virus-induced silencing (BSMV-VIGS) method was employed to acquire *TaMCA-Id-*silenced seedlings, which were used to explore the mechanism of interaction between autophagy and *TaMCA-Id* in the process of NaCl stress-triggered PCD. The results may help to clarify the molecular components involved in plant PCD and autophagy, especially under salt stress conditions.

## Materials and Methods

### Wheat Seedling Growth and Stress Conditions

Jimai22, a salt-tolerant variety, was acquired from the Tianjin Academy of Agricultural Sciences (China) (Dou et al., 2021). Jimai 22 is also a high-yield, multi-resistant, and high-quality wheat variety with medium gluten. Wheat seedlings were cultured by hydroponics as previously described (Yue et al., 2018, 2021a). At the two-leaf stage, healthy seedlings at the same growth rate were chosen and exposed to the same one-fourth strength Hoagland's solution added with 150 mM NaCl, and the seedlings grown in the one-fourth strength Hoagland's solution without NaCl were set as the controls. After 48 h exposure to 150 mM NaCl, the leaves and about 2 cm root tips of the control and treated group were separately collected from at least five seedlings and placed into liquid nitrogen for the extraction of RNA, which used for the following gene cloning and quantitative real-time PCR (qPCR) assay.

### Cloning, Sequencing, and Phylogenetic Analysis of *TaMCA-Id*

Total RNA in the wheat root or leaf samples was extracted with TRIzol reagent (Invitrogen, USA). Then, reverse transcription kits (Promega, USA) were employed to reverse transcribe RNA into first-strand cDNA. The cDNA was used as a template in the following PCR assay. A unigene (accession no. TraesCS4D02G358100) released from EnsemblPlants was chosen and cloned, which was conferred to wheat seedlings' tolerance to salt stress in an RNA-Seq assay (Yue et al., 2021b). Its complete coding sequence (CDS) was amplified from the first-strand cDNA using TransStart Fast Pfu DNA polymerase (TransGen, China), with a specific pair of primers presented in Supplementary Table 1. The procedure of PCR followed the manufacturer's instructions. After poly(A) tailing, the PCR products were cloned into the vector of pGEM^®^-T Easy (Promega, USA) and then sequenced by Sangon Biotech (Shanghai, China). The molecular weight (Mw) and theoretical isoelectric point (pI) of the putative *TaMCA-Id* were assessed using ExPASy software (http://www.expasy.org/). The homology search proteins using the deduced amino acid sequence of *TaMCA-Id* were carried out according to BLASTp program of the National Center for Biotechnology Information (NCBI) (http://www.ncbi.nlm.nih.gov/). The multiple sequence alignment was conducted with Bioedit software. The functional and conserved domains were predicted with Smart software (http://smart.embl-heidelberg.de/smart/set_mode.cgi). The maximum likelihood phylogenetic tree was built with MEGA-X software.

### Subcellular Localization of *TaMCA-Id*

The open reading frame of *TaMCA-Id* without termination codon was amplified from the first-strand cDNA or vector of pGEM^®^-T Easy-TaMC1. Then, the amplification products were digested with XbaI and BamHI and were subcloned into the *pAN583*:*mCherry* vector with pEASY^®^-Basic Seamless Cloning and Assembly Kit (TransGen Biotech, China), generating the *TaMC1*-*mCherry* fusion construct pAN583:TaMC1-mCherry. Then, the *pAN583*:*TaMC1*-*mCherry* fusion construct and *pAN583*:*mCherry* (control) were transferred into wheat protoplasts, respectively, with PEG-mediated transformation, as described by Li et al. (2018). After incubated for 20 h at 25°C, the mCherry signals in the transformed wheat protoplast were detected with a confocal laser scanning microscope (Nikon Ti2 Eclipse A1). The primers were presented in Supplementary Table 1.

### BSMV-VIGS Assay

A cDNA fragment of 170 bp (+512 bp to +680 bp) was employed to acquire *TaMCA-Id* silenced vector. After this fragment was inserted in *BSMV*γ plasmid (*BSMV*γ*:TaMCA-Id*), the plasmids of *BSMV*α, *BSMV*γ*:GFP*, and *BSMV*γ*:TaMCA-Id* were linearized with *Mlu I* (Takala, Dalian, China). *BSMV*β was linearized with *Spe I* (Takala, Dalian, China). Then, RiboMAX largeScale RNA Production-T7 kit (Promega, USA) was used to *in vitro* transcribe these linearized plasmids. Ribom 7G Cap Analog (Promega, USA) was employed to produce the 5′-capped BSMV RNA molecules for following *BSMV*-*VIGS* inoculated experiments. These experiments (containing vector construction, *in vitro* transcription, *BSMV-VIGS* inoculation, *TaMCA-Id*-silenced seedlings identification, and silence efficiency assessment of *TaMCA-Id* were conducted as previously described (Yue et al., 2021a). For salt stress, part of the *BSMV-VIGS*-incubated seedlings with three expanded leaves were exposed to 150 mM NaCl treatment for 6 days. All experiments were repeated at least three times, and 20 seedlings were treated for each repeat. The used primes are present in Supplementary Table 1.

### Physiological Measurements and Histochemical Staining

After 150 mM NaCl was stressed for 6 days, the control and *TaMCA-Id*-silenced seedlings were used to carry out the following experiment. The *in situ* generation of H_2_O_2_ and ${\text{O}}_{2}^{.-}$ in wheat leaves was detected using 3,3-diaminobenzidine (DAB) and nitro blue tetrazolium (NBT), respectively. The staining procedures were carried out as previously described (Yue et al., 2021a). The quantitative determination of H_2_O_2_ and ${\text{O}}_{2}^{.-}$ was performed following a previous study (Yue et al., 2021b). Evans blue staining was carried out to visualize the degree of cell damage in the wheat leaves under NaCl treatment. The leaves samples were immersed in 0.25% Evans blue staining solution and stained for 24 h in darkness. Then, the tissues were rinsed with decolorization buffer (absolute ethanol and glycerol, 9:1, v/v) until they became colorless. Then, the leaves were photographed by stereoscopic microscope (Nikon C-fled2). Monodansylcadaverine (MDC) was used to label autophagosomes (Khalid et al., 2019). The observation of autophagosomes in wheat leaves was carried out according to our previous research (Yue et al., 2021a).

The activities of peroxidase (POD), superoxide dismutase (SOD), and catalase (CAT) were analyzed as described by Yue et al. (2021a). For determination of the caspase-3 activity, the leaf tissues (0.1 g) were ground with liquid nitrogen and then were immersed in the lysis buffer (150–200 μl) according to the Caspase-3 Colorimetric Assay Kit manufacturer's guidance (Cat#: KGA202, KeyGEN BioTECH, China). Then, homogenate collection and detection of the supernatant protein concentration and determination of caspase-3-like activity were performed following the manufacturer's instructions.

### qPCR Assay

Total RNA extraction of the leaf samples, first-strand cDNA transcription, and qPCR assay were conducted as previously described (Yue et al., 2018, 2021a). The involved primers are present in Supplementary Table 1.

### Determination of Chlorophyll Fluorescence Parameters

The representative chlorophyll fluorescence parameters, containing PSII photochemistry (Fv/Fm), quantum yield of PSII (Y(II)), quenching coefficient (qP), electron transfer rate (ETR), and non-photochemical quenching coefficient (NPQ), were examined according to our previous study (Yue et al., 2021a).

### Statistical Analyses

Every experiment was performed at least three repetitions. The data were present as mean ± SD. All statistical analyses were carried out according to Duncan's multiple range tests with SPSS software. *P* < 0.05 indicated significant differences.

## Results

### Cloning and Characterization of the Wheat MC Gene *TaMCA-Id*

**Figure 1:**
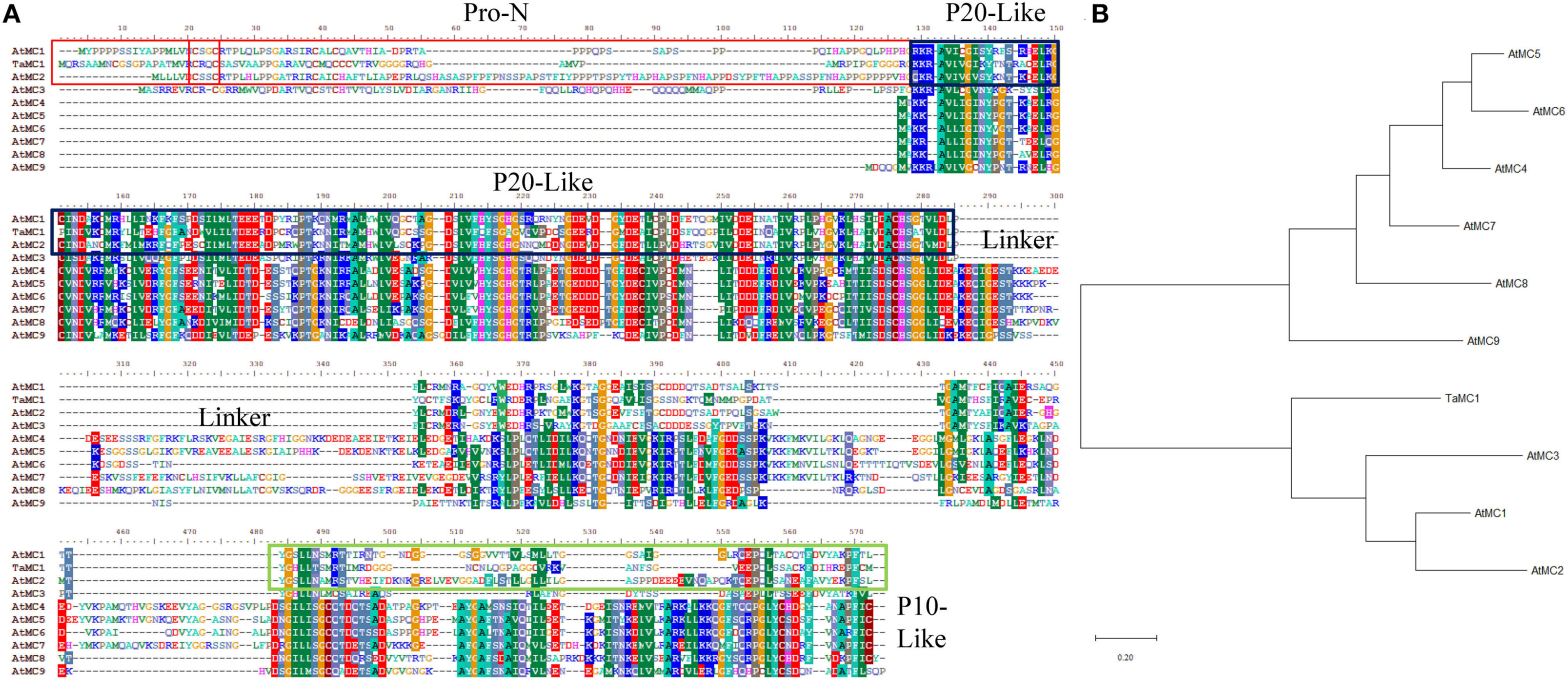
Sequence analysis of *TaMCA-Id*. **(A)** Multi-sequence alignment of *TaMCA-Id* with other metacaspase proteins from *Arabidopsis*. Amino acid sequences from 10 metacaspase share similar structures, containing Pro-N domain, P20-like domain, Linker, and P10-like domain. *Ta, Triticum aestivum* L.; *At, Arabidopsis thaliana*. **(B)** Phylogenetic analysis of *TaMCA-Id*. *TaMCA-Id* and function-known metacaspase in *Arabidopsis thaliana* (At) are used to construct their phylogenetic tree using MEGA-X software. TaMC1 in the figure indicates TaMCA-Id. AtMC1 to AtMC9 in the figure indicate AtMCA1 to AtMCA9, respectively.

After amplification, the complete coding sequence (CDS) of the salt-responsive unigene (accession no. TraesCS4D02G358100) was acquired. Sequence analysis showed that the open reading frame *TaMCA-Id* was 1,071 bp in length, which encoded 356 amino acid residues with molecular weight and isoelectric point of 38,337.03 Da and 8.45, respectively. It was located on chromosome 4D. According to Chen et al. (2020), *TaMCA-Id* was used as a probe to search for homologs in the Triticeae-GeneTribe database. TraesCS5A02G548200, located on chromosome 5A, TraesCS4B02G381800, located on the chromosome 4B, and TraesCS4D02G358800, located on the chromosome 4D, were identified as *TaMCA-Id* homologs in wheat. They encode peptide sequences of 299, 352, and 346 amino acids, respectively. The amino acid residues of the above three homologs and *TaMCA-Id* share identities of 85–94% (Supplementary Figure 1). *TaMCA-Id* protein had a putative conserved Peptidase_C14 domain with caspase-like catalytic activity (Supplementary Figure 2). Multi-sequence alignments of *TaMCA-Id* sequence and nine MCs of *Arabidopsis thaliana* indicated that *TaMCA-Id* contained a typical N-terminal pro-domain that was a specific characteristic of type I MCs, and there was also a linker region between the p20 and p10 subunits that was a specific property of Type II MCs (Figure 1A). However, the phylogenetic tree revealed that the phylogenetic relationship of *TaMCA-Id* was related to AtMCA1, which belonged to type I MCAs (Figure 1B). Based on the above bioinformatics analysis and Minina et al. (2020) study, the amplified gene was eventually named *TaMCA-Id*.

### TaMCA-Id Responds to NaCl Treatment

**Figure 2:**
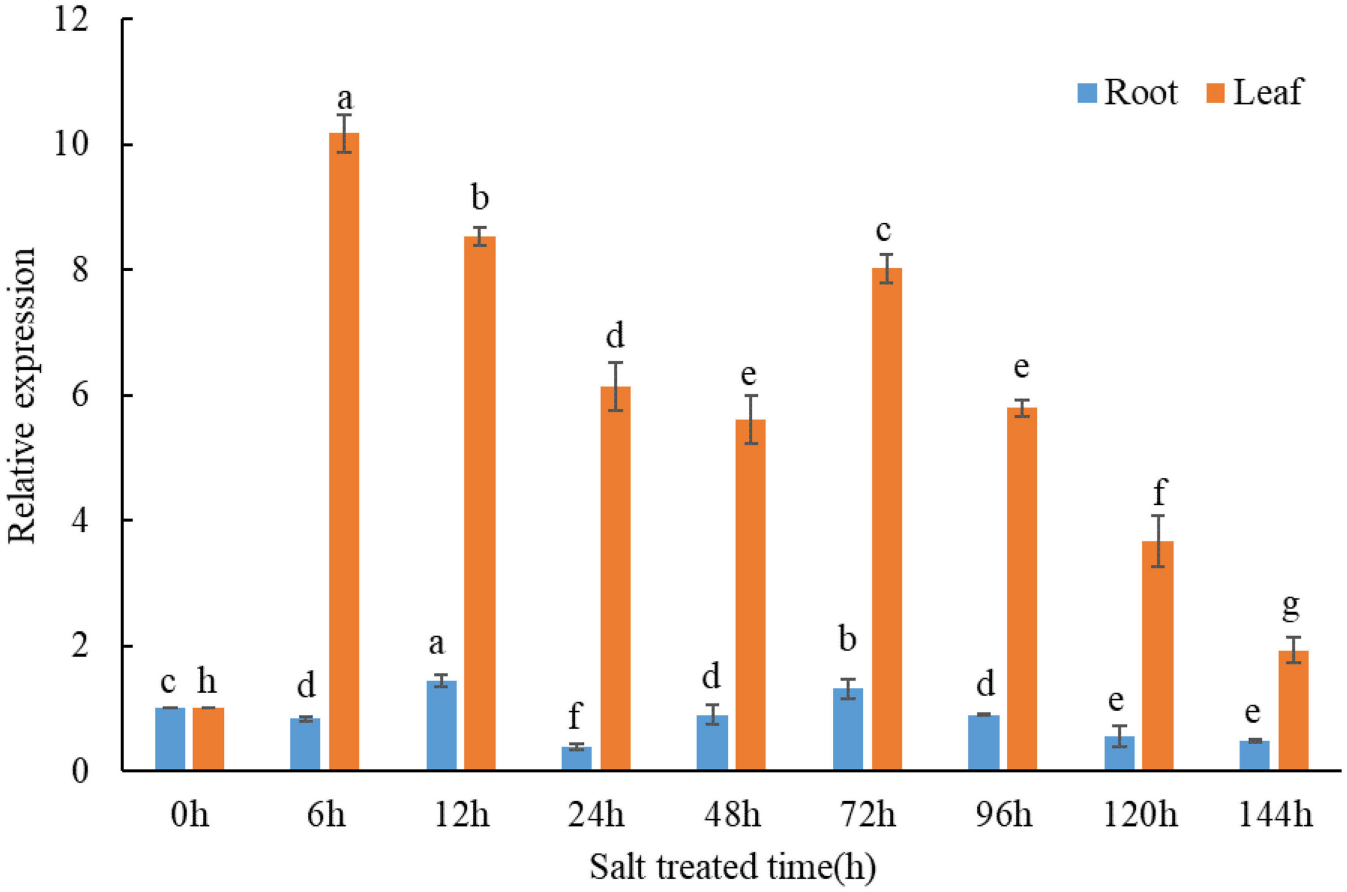
Transcript levels of *TaMCA-Id* in roots and leaves of wheat seedlings under NaCl treatment using qPCR assay. Data are presented as mean ± SD from at least three independent experiments. Bars with different letters are significantly different at *P* < 0.05.

qPCR assays were conducted to investigate the expression response of *TaMCA-Id* to a time-series NaCl treatment in wheat seedlings. The result indicated that *TaMCA-Id* expression in the roots and leaves was significantly upregulated under NaCl treatment (Figure 2). The results proved that *TaMCA-Id* was involved in the NaCl stress response of wheat seedlings.

### Subcellular Localization of *TaMCA-Id*

**Figure 3:**
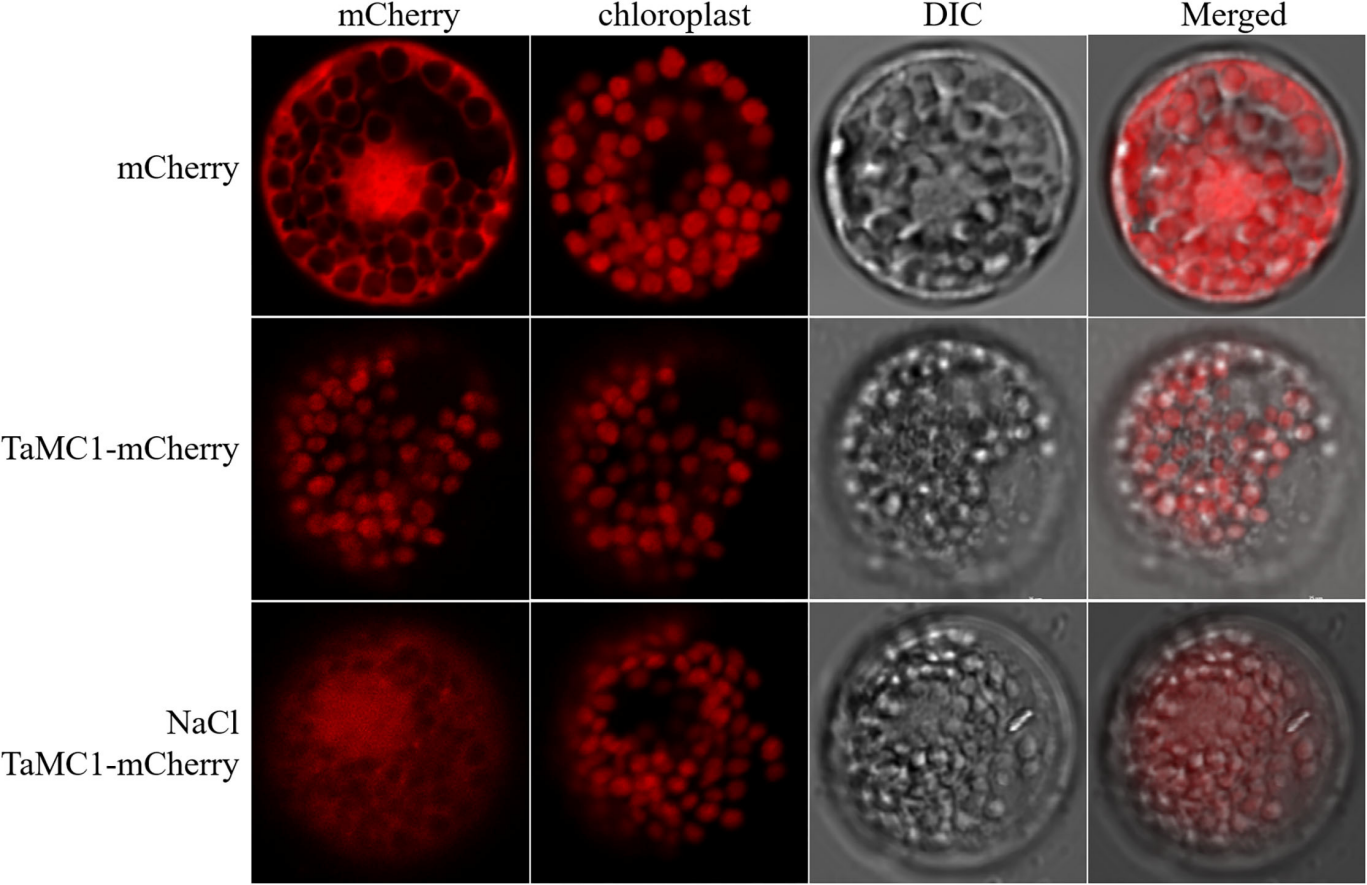
Subcellular localization of *TaMCA-Id*. Wheat protoplasts were transformed with *pAN583*:*mCherry* or *GFP*:*mCherry via* the polyethylene glycol (PEG)-mediated method. *TaMCA-Id* was mainly localized to the chloroplast in wheat protoplasts, and exhibited nucleocytoplasmictrafficking under NaCl treatment. TaMC1 in the figure indicates TaMCA-Id.

mCherry and *TaMCA-Id*-mCherry were each expressed in wheat mesophyll protoplasts to investigate the localization of TaMCA-Id. As shown in Figure 3, under normal conditions for the maintenance of transfected protoplasts, the fluorescence signals of mCherry spread in the whole cell, including the nucleus, while those of *TaMCA-Id*-mCherry mainly spread in the chloroplast. Under NaCl treatment, some of this *TaMCA-Id*-mCherry fusion protein was tracked in the nucleus or in the cytoplasm (Figure 3). These features may ensure that *TaMCA-Id* functions effectively in signal transduction under NaCl stress in wheat seedling leaves.

### Silencing of *TaMCA-Id* Increases Salt Sensitivity of Wheat Seedlings

To better investigate the function of *TaMCA-Id* in wheat response to NaCl stress, we studied *TaMCA-Id* loss of function in wheat seedlings by conducting *BSMV*-*VIGS*-inoculated experiment. After the third and newly grown leaves of the *BSMV*-*VIGS*-inoculated seedlings showed virus infection-induced chlorosis, at least three seedlings were randomly selected to examine the silencing efficiency of *TaMCA-Id*. Compared with *BSMV*-*VIGS*-*GFP*-inoculated seedlings, the expression of *TaMCA-Id* was significantly suppressed in the leaves of *TaMCA-Id*-silenced seedlings (Supplementary Figure 3).

Under non-stressed conditions, *TaMCA-Id* silencing slightly affect root length and the third leaf length of wheat seedlings, while *BSMV*-*VIGS* inoculation produced a little decrease in the root length and the third leaf length compared with that in the wide-type (WT) seedlings (Supplementary Table 2). After exposure to NaCl treatment for 6 days, silencing of *TaMCA-Id* aggravated the NaCl stress-induced damage to wheat seedling growth (Supplementary Figure 4), with shorter lengths of both roots and the third leaves (Supplementary Table 2). The root length of *TaMCA-Id*-silenced seedlings and *BMSV-VIGS-GFP*-inoculated seedlings was decreased by 29% and 24% as compared with the controls, respectively. The leaf length of *TaMCA-Id*-silenced seedlings and *BMSV-VIGS-GFP*-inoculated seedlings was decreased by 28% and 26% as compared with the controls, respectively. The above results suggested that silencing of *TaMCA-Id* increased wheat seedling sensitivity to NaCl treatment.

### Silencing of *TaMCA-Id* Enhances the Autophagy Activity in Wheat Leaves at the Seedling Stage Under NaCl Treatment

**Figure 4:**
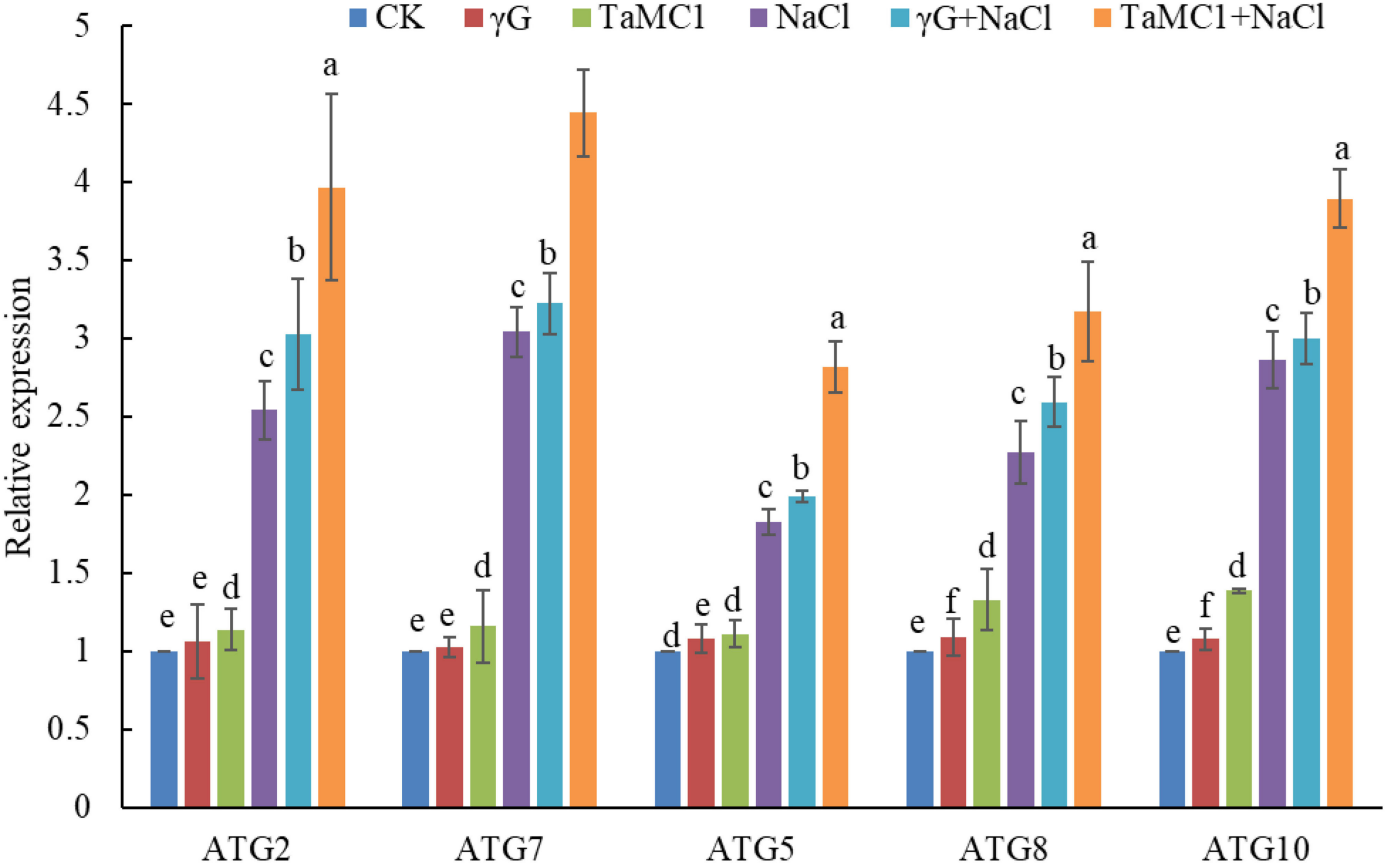
The effect of silencing of *TaMCA-Id* on the relative expression analysis of key *ATGs* in leaves of wheat seedlings under NaCl stress. CK was the wild-type wheat seedlings. γG was the *BSMV-VIGS-GFP*- inoculated wheat seedlings. *TaMC1* was the *BSMV-VIGS-TaMCA-Id*- inoculated wheat seedlings. Data are shown as mean ± SD (*n* = 3) of three independent experiments. The different letters in each treatment show the significant difference (*P* < 0.05).

After exposure to NaCl treatment for 6 days, silencing of *TaMCA-Id* promoted autophagy in wheat seedling leaves, with upregulation of the key *ATGs*, including *ATG2, ATG5, ATG7, ATG8*, and *ATG10* (Figure 4), and obvious increase of autophagosomes in wheat leaves (Supplementary Figure 5), as compared with those in the *BSMV*-*VIGS*-*GFP*-inoculated seedlings and the WT seedlings. These results implied that there was an interaction between autophagy and *TaMCA-Id* silencing in the regulating NaCl stress response of wheat seedlings.

### Silencing of *TaMCA-Id* Promotes NaCl Stress-Triggered PCD in Leaves of Wheat Seedlings

**Figure 5:**
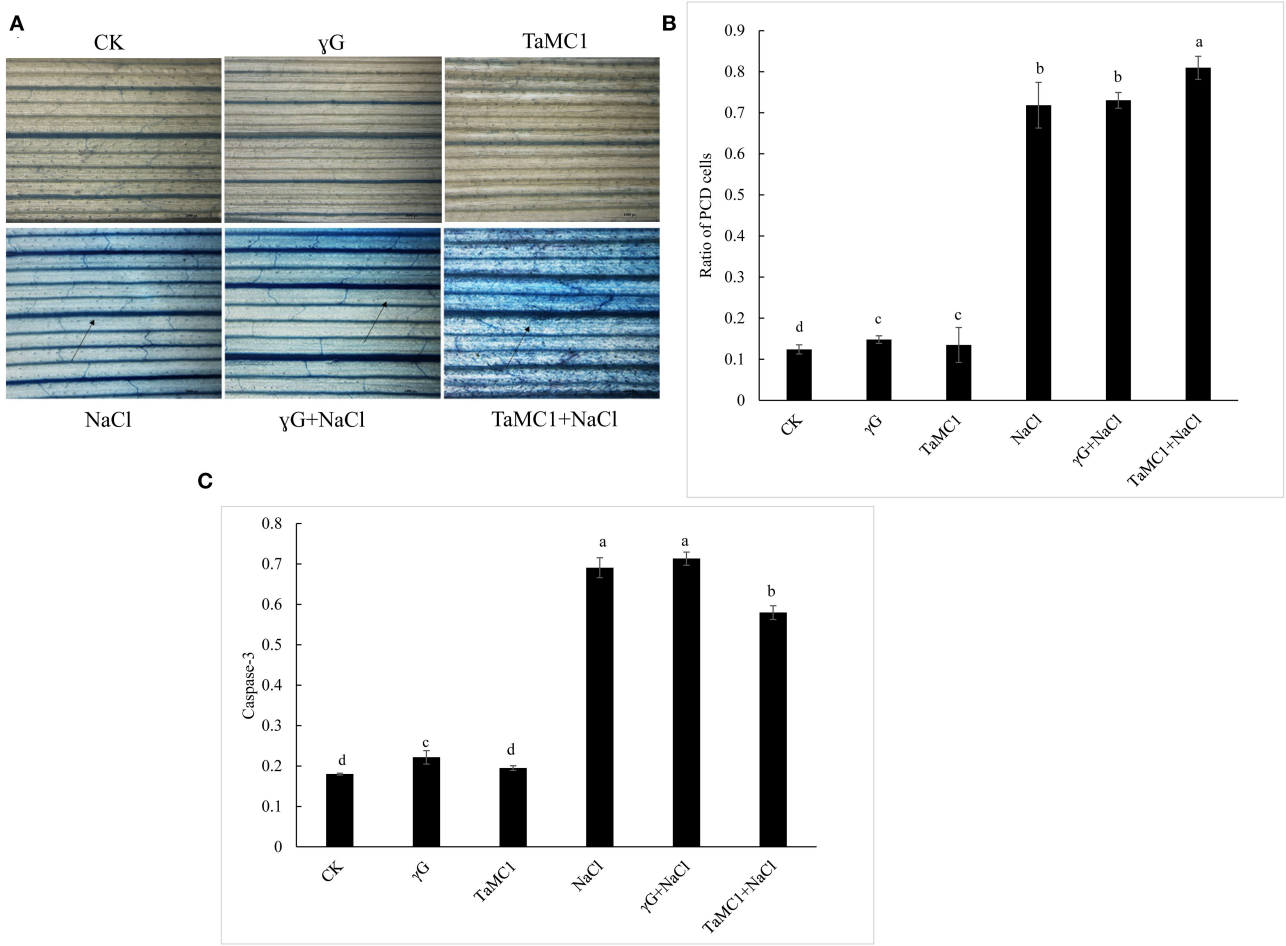
The effect of silencing of *TaMCA-Id* on cell death in leaves of wheat seedlings under NaCl stress. CK was the wild-type wheat seedlings. γG was the *BSMV-VIGS-GFP*- inoculated wheat seedlings. *TaMC1* was the *BSMV-VIGS-TaMCA-Id*- inoculated wheat seedlings. **(A)** Evans blue staining results. **(B)** The ratio of PCD cells in wheat leaves determined with Tunel assays. Standard deviations are shown (*n* > 3, ±SD, *P* < 0.05). The different letters in each treatment show the significant difference (*P* < 0.05). **(C)** The effect of silencing of *TaMCA-Id* on the caspase-3-like activity in leaves of wheat seedlings under NaCl stress. Standard deviations are shown (*n* > 3, ±SD, *P* < 0.05). The different letters in each treatment show the significant difference (*P* < 0.05).

To further investigate whether *TaMCA-Id* silencing was involved in the cell death caused by NaCl treatment, Evans blue was used to *in situ* stain the wheat seedling leaves. The results showed that the cell death caused by NaCl treatment was obvious in wheat seedling leaves, and silencing *TaMCA-Id* aggravated NaCl stress-induced cell death in the leaves of wheat seedlings (Figure 5A). TUNEL assay was performed to determine whether PCD was involved in wheat response to NaCl stress. The results showed that NaCl treatment enhanced the ratio of TUNEL-positive PCD cells in wheat seedling leaves. Silencing of *TaMCA-Id* further elevated the NaCl stress-induced PCD ratio in wheat seedling leaves (Figure 5B). These results implied that *TaMCA-Id* had a significant regulatory effect on the PCD level in wheat seedling leaves under NaCl treatment.

Colorimetric experiment was employed to detect whether *TaMCA-Id* functioned in regulating caspase-3-like activity in wheat seedling leaves under NaCl treatment. After exposure to NaCl treatment for 6 days, silencing of *TaMCA-Id* triggered a higher NaCl stress-induced caspase 3-like activity in wheat seedlings leaves, indicating that *TaMCA-Id* probably acted as a regulator of caspase 3-like activity (Figure 5C). Taken together, these results suggested that *TaMCA-Id* functioned in promoting PCD in wheat leaves at the seedling stage under the treatment of NaCl.

### Silencing of *TaMCA-Id* Reduces the Antioxidant Capacity of Wheat Seedling Leaves Under NaCl Treatment

**Figure 6:**
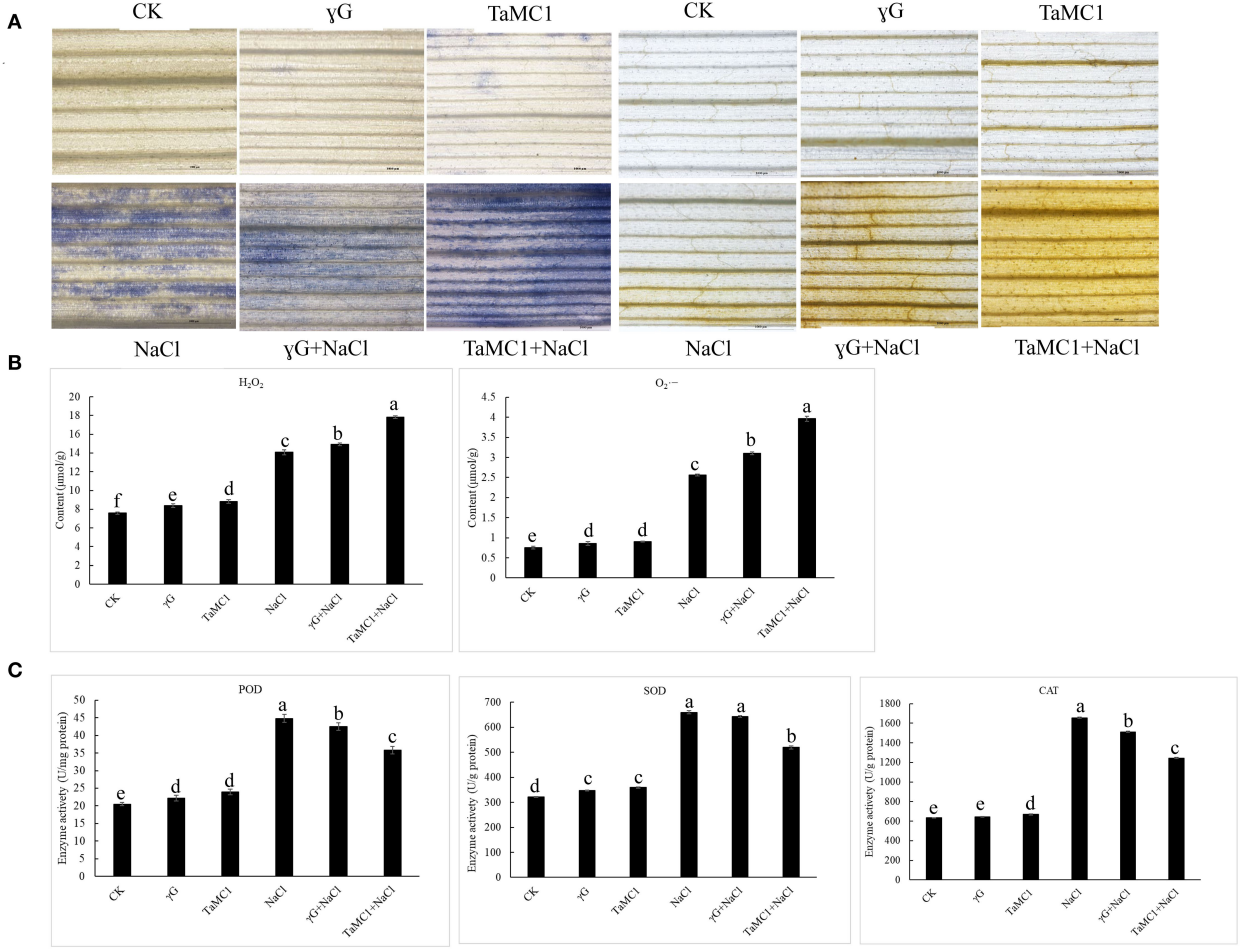
The effect of silencing of *TaMCA-Id* on the ROS accumulation in leaves of wheat seedlings under NaCl stress. CK was the wild-type wheat seedlings. γG was the *BSMV-VIGS-GFP*- inoculated wheat seedlings. *TaMC1* was the *BSMV-VIGS-TaMCA-Id*- inoculated wheat seedlings. **(A)** NBT staining of the generation and accumulation of ${\text{O}}_{2}^{.-}$ in leaves of wheat seedlings, and DAB staining of the generation and accumulation of H_2_O_2_ in leaves of wheat seedlings. **(B)** The content of ${\text{O}}_{2}^{.-}$ and H_2_O_2_ in leaves of wheat seedlings. **(C)** The activities of POD, SOD, and CAT in leaves of wheat seedlings. Standard deviations are shown (*n* > 3, ± SD, *P* < 0.05). All the experiments presented in this study were performed at least three times, and similar results were obtained.

To study the influence of *TaMCA-Id* on the antioxidant capacity of wheat leaves under NaCl treatment, the ROS levels and the activities of some antioxidant enzymes were monitored. Under non-stress conditions, there were no obvious changes in the accumulation of ${\text{O}}_{2}^{.-}$ and H_2_O_2_ between *BSMV-VIGS-GFP*-inoculated and WT seedlings. NaCl stress significantly elevated the production of ${\text{O}}_{2}^{.-}$ and H_2_O_2_ in the wheat leaves. Silencing of *TaMCA-Id* significantly kept the content of H_2_O_2_ and ${\text{O}}_{2}^{.-}$ in wheat seedling leaves at higher levels under NaCl treatment (Figure 6A). The quantitative analysis results of H_2_O_2_ and ${\text{O}}_{2}^{.-}$ concurred with the staining results (Figure 6B). After 6 days of NaCl treatment, the activities of POD, SOD, and CAT in wheat seedlings were elevated. Silencing of *TaMCA-Id* decreased the activities of POD, SOD, and CAT in the wheat leaves under NaCl treatment (Figure 6C). These results indicated that the *TaMCA-Id* silencing promoted ${\text{O}}_{2}^{.-}$ and H_2_O_2_ generation in wheat seedling leaves by impairing antioxidant capacity under NaCl treatment.

### Silencing of *TaMCA-Id* Aggravates the NaCl Stress-Induced Injury of Photosystem II (PSII)

Compared with the control, NaCl stress significantly decreased Fv/Fm, Y(II), qP, and ETR and increased NPQ of wheat seedlings. After 6 days of NaCl treatment, silencing of *TaMCA-Id* further decreased Fv/Fm, Y(II), qP, and ETR and increased NPQ of wheat seedlings compared with those of the *BSMV-VIGS-GFP*-inoculated and WT seedlings (Table 1). These results indicated that the inhibition of *TaMCA-Id* expression aggravated NaCl treatment-induced PSII injury in wheat seedlings.

**Table 1:** The changes in chlorophyll fluorescence parameters in leaves of *BSMV*-*VIGS*-*GFP*- (γG), *BSMV*-VIGS-*TaMCA-Id- (TaMC1)*-inoculated wheat seedlings and the wild-type seedlings (WT) under NaCl stress.

**Treatments**	**Fv/Fm**	**Y(II)**	**qP**	**NPQ**	**ETR**
WT	0.79 ± 0.03^a^	0.56 ± 0.00^a^	0.74 ± 0.01^a^	0.55 ± 0.00^e^	58.16 ± 0.08^a^
γG	0.78 ± 0.04^a^	0.54 ± 0.01^b^	0.74 ± 0.01^a^	0.56 ± 0.01^de^	57.98 ± 0.07^a^
TaMC1	0.78 ± 0.00^a^	0.53 ± 0.01^b^	0.73 ± 0.01^a^	0.57 ± 0.02^d^	57.24 ± 0.04^b^
WT+NaCl	0.73 ± 0.00^b^	0.34 ± 0.02^c^	0.54 ± 0.00^b^	1.20 ± 0.02^c^	40.68 ± 0.04^c^
γG+NaCl	0.73 ± 0.00^b^	0.33 ± 0.01^c^	0.53 ± 0.00^b^	1.22 ± 0.01^b^	40.16 ± 0.05^c^
TaMC1+NaCl	0.71 ± 0.01^c^	0.31 ± 0.00^d^	0.51 ± 0.02^c^	1.48 ± 0.03^a^	37.56 ± 0.05^d^

*The data are shown as mean ± SD (n = 3) of three independent experiments. The data with different letters in same column show significant difference (P < 0.05)*.

## Discussion

Salinity stress has a damaging impact on plant growth and threatens agronomical production (Signorelli et al., 2019). To counteract this, plants have developed many strategies for plant adaptability to salt stress. Among them, both autophagy and PCD are evolutionarily conserved strategies for plants to resist various environmental stresses (Chen et al., 2021; Ren et al., 2021). Crosstalk between PCD and autophagy as a whole at “process level” is very complex, which is regulated by cysteine proteases (animal caspases and plant MCAs; Minina et al., 2014). Under stress conditions, as a pro-survival mechanism, autophagy is always initiated to combat PCD *via* eliminating excess ROS-triggered oxidative damage and being conducive to cell survival (Yue et al., 2021a). For example, Zhou et al. (2018) indicated that tobacco mosaic virus (TMV) local inoculation activated PCD and autophagy in tomatoes, and ROS was the signaling molecule that promoted autophagy in root-tip cells of the tomato plant. Under waterlogging stress, autophagy can be induced to inhibit PCD, which maintained wheat seedlings' survival, with the increased activities of MCAs and caspase-like protease (Zhou et al., 2021). Our previous study confirmed that autophagy was induced by NaCl stress to improve the adaptability to NaCl treatment in wheat seedlings *via* negatively regulated salt-induced PCD. This process was accompanied with differential expression of *TaMCA-Id* in leaves of wheat seedlings, and silencing of *ATG2* or *ATG7* triggered higher or lower expression of different *TaMCA-Id* and significantly promoted PCD in wheat roots and leaves at the seedling stage under NaCl treatment (Yue et al., 2021a). These results suggest that there is an interplay between autophagy and PCD, which ensures the tight regulation of cellular homeostasis under stress conditions, and MCAs function in regulating the autophagy-PCD crosstalk induced by stress treatment. But the underlying regulating mechanisms are less clear.

The *MCAs* act to regulate plant growth, development, and multiple stress responses (Gong et al., 2020; Roohollah et al., 2020). This study acquired a novel *TaMCA-Id* that had typical characteristics of type I MCA family, which could contribute to the regulation of NaCl-caused PCD and activity of caspase 3-like proteases in wheat seedlings. The subcellular localization of *TaMCA-Id* was mainly in the chloroplast and was transferred from chloroplast to cytoplasm and nucleus induced by NaCl stress. The result was consistent with Castillo-Olamendi et al. (2008), which showed that an AtMCP1b was localized in the chloroplasts. Hao et al. (2016) suggested that Type I TaMCA1 was localized in the cytoplasm and mitochondria. Interestingly, *Arabidopsis* MCA9 was located in the apoplast, nucleus, and cytoplasm, and its subcellular localization was altered in the late autolysis process (Vercammen et al., 2004, 2006). In this study, the *TaMCA-Id* nucleocytoplasmictrafficking under NaCl stress might be important for PCD induction. The above results suggest that MCAs localize in different cellular compartments implying their functional diversification.

Consequently, we suggested that *TaMCA-Id* might play a vital role in the NaCl stress response of wheat seedlings, as a positive cell death regulator. We further carried out a knockdown of *TaMCA-Id* to confirm its function in the NaCl stress response of wheat seedlings. Chlorophyll fluorescence reflects PSII activity, which represents plant damage (He et al., 2018; Goussi et al., 2018). We found that silencing of *TaMCA-Id* damaged PSII and promoted NaCl-induced PCD in wheat seedling leaves under NaCl treatment. The result was consistent with a previous study that suggested *TaMCA1* could suppress cell death caused by the mouse *Bax* gene in *N. benthamiana* and wheat leaves, and it could be a negative cell death regulator function in regulating wheat-*Pst* interaction (Hao et al., 2016). However, this result is different from the results that *AhMCA1* positively regulated PCD in peanut root tips triggered by Al treatment (Yao et al., 2020). Interestingly, silencing of *TaMCA-Id* also promoted NaCl-induced autophagy in the leaves of wheat seedlings. A similar phenomenon was observed in *Arabidopsis* vascular xylem differentiation, and the vascular tracheary elements (TE) cell type presented even higher autophagic activity triggered by the inhibition of the TE-specific *MCA9* using RNAi, which was detrimental to the surrounding cells (Escamez et al., 2016). These results implied that *TaMCA-Id* might function prior to cell death. Notably, our previous study suggested that *ATG2-* or *ATG7*-suppressed autophagy led to the upregulation of *TaMCA-Id* in wheat leaves at the seedling stage under NaCl treatment (Yue et al., 2021a). Taken together, these results showed that autophagy was activated by the inhibition of *TaMCA-Id* expression, which could initially maintain cellular homeostasis under NaCl treatment, through removing and recycling damaged proteins and organelles. There is a complex interaction between autophagy and PCD. They were activated together and had mutual regulation relationship under NaCl stress. During NaCl stress, autophagy to a certain extent could inhibit PCD, and the increase of PCD level with the treatment time extended also could lead to enhanced autophagy and eventually follow autophagic cell death, which is a non-apoptotic form of PCD, due to stress-induced nutrient deficiency.

Salt stress leads to the ROS burst in plants, which is a necessary event in plants' salt stress response (Zhang et al., 2019). A growing number of evidence indicated the function of ROS in the upstream of autophagy and PCD (Demidchik et al., 2018; Zhou et al., 2018a; Wang et al., 2020). Similarly, our study found that silencing of *TaMCA-Id* further enhanced ROS production induced by NaCl treatment in wheat leaves at the seedling stage. Eventually, the excess ROS might promote autophagy and PCD in leaves of wheat seedlings under NaCl treatment. Thus, we deduced that *TaMCA-Id* could contribute to the regulation of autophagy and PCD *via* ROS levels. Plants have invoked the antioxidant defense system, including SOD, CAT, POD, and other enzymes, to prevent oxidative damage (Buttar et al., 2020). Our results showed that silencing of *TaMCA-Id* suppressed the antioxidant defensive response to destroy the ROS scavenging system and thus resulted in excess ROS accumulation under NaCl treatment. The transcriptional levels of cell death- and defense-related genes that determine cell fate under NaCl treatment, including calcium-dependent protein kinases (CDPKs), containing *CDPK21* and *CDPK27*, cysteine-rich receptor-like protein kinases (CRKs), containing *CRK10* and *CRK26, glyceraldehyde-3-phosphate dehydrogenase B (GAPD), MYB, NAC, cytochrome P450 94A2-like (CYP94A2)*, and *respiratory burst oxidase homolog protein B-like (Rboh-like)*, were also changed in the leaves of *TaMCA-Id*-silenced seedlings compared with that in control seedlings (Supplementary Figure 6).

## Conclusion

Autophagy and PCD are the salt tolerance strategies employed by wheat seedlings. They controlled the cellular homeostasis through eliminating damaged macromolecules and organelle, or unnecessary cells under NaCl stress. *TaMCA-Id* is mainly localized in the chloroplast and exhibits nucleocytoplasmictrafficking under NaCl stress. *TaMCA-Id* would be a regulator of stress-induced cell death through regulating ROS-induced autophagy. In a word, silencing of *TaMCA-Id* was able to reduce NaCl tolerance of wheat seedlings *via* promoting ROS generation which further took part in the regulation of autophagy and PCD triggered by NaCl treatment. This study suggests that there is an interplay between autophagy and PCD, which ensures the tight regulation of cellular homeostasis under stress conditions, and MCAs function in regulating the autophagy-PCD crosstalk induced by stress treatment.

## Data Availability Statement

The raw data supporting the conclusions of this article will be made available by the authors, without undue reservation.

## Author Contributions

J-yY: methodology, validation, formal analysis, writing (original draft), and funding acquisition. Y-jW: methodology, validation, and software. J-lJ and W-wW: visualization and software. H-zW: writing, reviewing, and editing the manuscript and supervision. All authors contributed to the article and approved the submitted version.

## Funding

This study was supported by the National Science Foundation of China (Nos. 31501234 and 31971829) and the Youth Talent Support Program of Tianjin Normal University (043135202RC1702).

## Conflict of Interest

The authors declare that the research was conducted in the absence of any commercial or financial relationships that could be construed as a potential conflict of interest.

## Publisher's Note

All claims expressed in this article are solely those of the authors and do not necessarily represent those of their affiliated organizations, or those of the publisher, the editors and the reviewers. Any product that may be evaluated in this article, or claim that may be made by its manufacturer, is not guaranteed or endorsed by the publisher.

## Abbreviations

MCAs: Metacaspases
PCD: programmed cell death
ATG: *autophagy-related gene*
BSMV-VIGS: barley stripe mosaic virus-induced silencing
VIGS: virus-induced gene silencing
ROS: reactive oxygen species
qPCR: quantitative real-time PCR
CDS: complete coding sequence
NCBI: National Center for Biotechnology Information
TMV: tobacco mosaic virus
TE: tracheary elements
O${\text{O}}_{2}^{.-}$: superoxide anion
H_2_O_2_: hydrogen peroxide
DAB, 3: 3-diaminobenzidine
NBT: nitro blue tetrazolium
SOD: superoxide dismutase
POD: peroxidase
CAT: catalase
PSII: photosystem II
Fv/Fm: PSII photochemistry
Y(II): quantum yield of PSII
qP: quenching coefficient
ETR: electron transfer rate
NPQ: non-photochemical quenching coefficient
qRT-PCR: real-time quantitative PCR
CDPKs: *calcium-dependent protein kinases*
CRK: *cysteine-rich receptor-like kinase*
GAPD: *glyceraldehyde-3-phosphate dehydrogenase B*
CYP94A2: *cytochrome P450 94A2-like*
Rboh-like: *respiratory burst oxidase homolog protein B-like*.
