# Supplementary material for: The Metacaspase TaMCA-Id Negatively Regulates Salt-Induced Programmed Cell Death and Functionally Links With Autophagy in Wheat
Source: Front Plant Sci. 2022 Jun 23;13:904933. doi: 10.3389/fpls.2022.904933 (PMC9260269; doi:10.3389/fpls.2022.904933)
Supplement: Supplementary file 1 [file Data_Sheet_1.pdf]

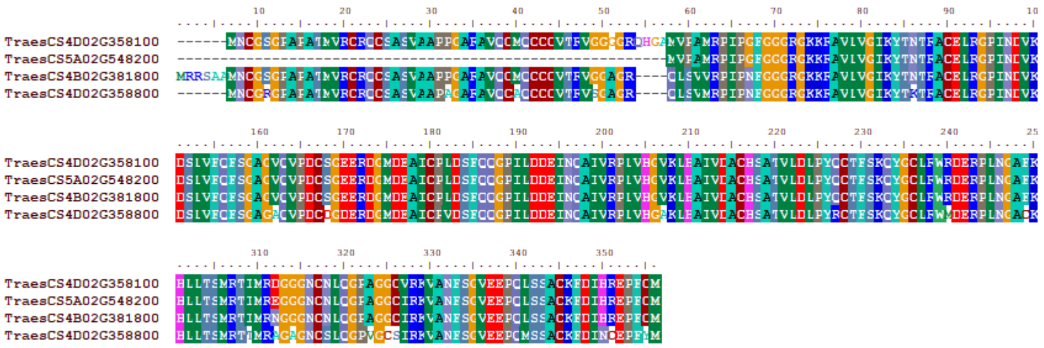

Supplementary Figure 1 Multi-sequence alignment of TaMCA-Id (TraesCS4D02G358100) with other three homologs in wheat.

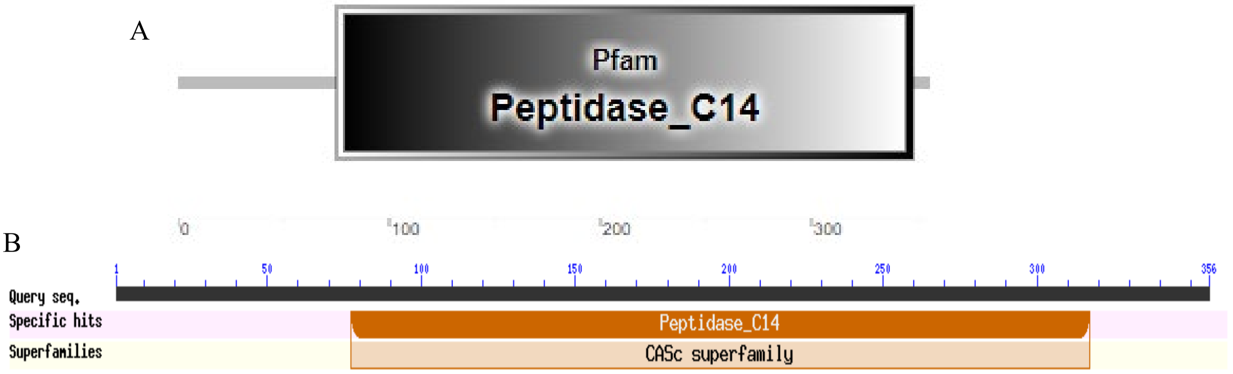

Supplementary Figure 2 Prediction of the conserved domain of TaMCA-Id

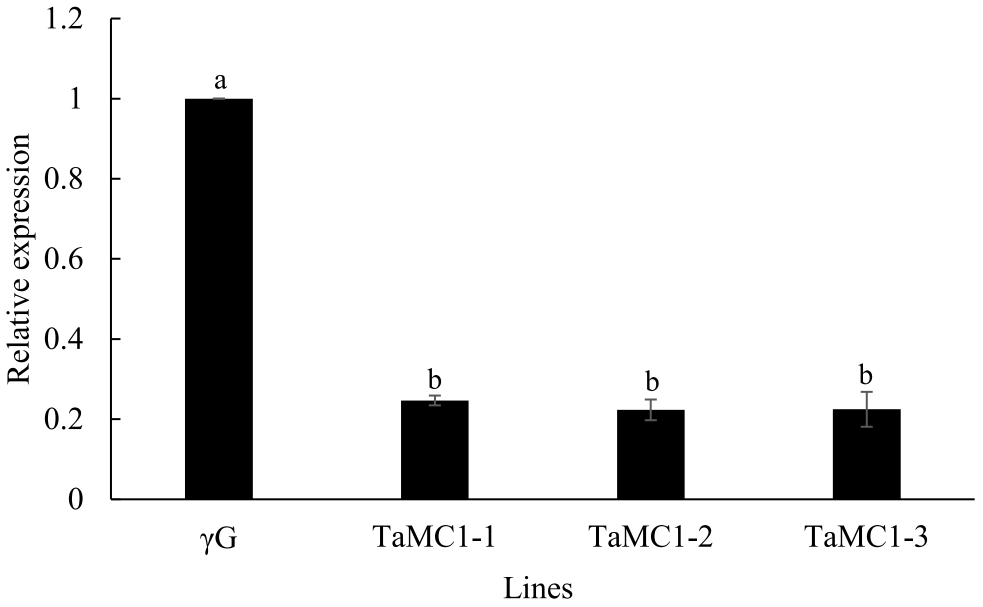

Supplementary Figure 3 Relative transcript levels of *TaMCA-Id* in leaves of *BSMV-VIGS*-inoculated wheat seedlings. The fourth leaves were sampled separately from *BSMV-VIGS*-inoculated wheat seedlings under normal conditions and used in the quantification of *TaMCA-Id* transcripts by qPCR. Amplification of the wheat *β-tubulin* gene served as an internal control. ɣG was from *BSMV-VIGS-GFP*-inoculated wheat seedlings and arabic numbers indicate individual *BSMV-VIGS-TaMCA-Id*-inoculated seedling. Bars with different letters are significantly different at $P < 0.05$.

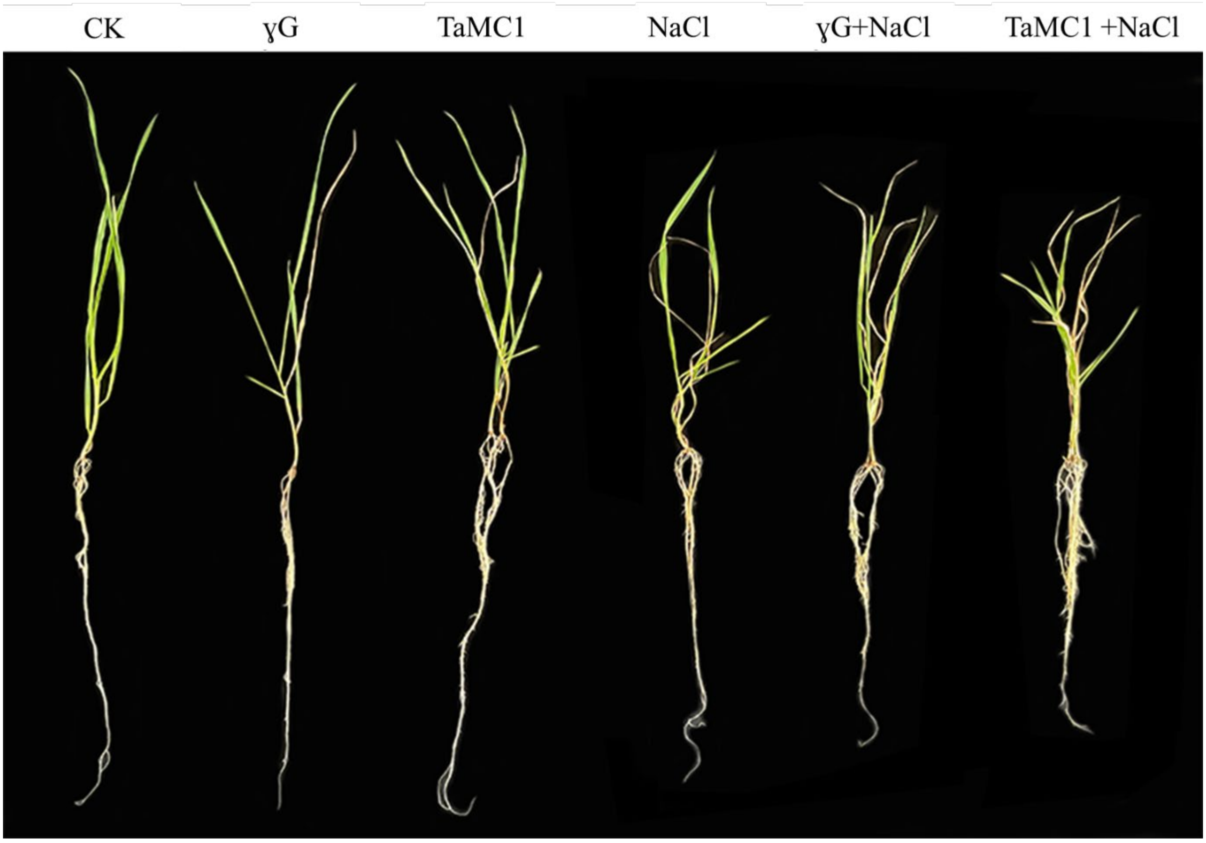

Supplementary Figure 4 The growth of *BSMV-VIGS-TaMC1-* and *BSMV-VIGS-GFP*-inoculated wheat seedlings under NaCl stress. CK was the wild-type wheat seedlings. γG was the *BSMV-VIGS-GFP*- inoculated wheat seedlings. TaMC1 was the *BSMV-VIGS-TaMCA-Id*- inoculated wheat seedlings.

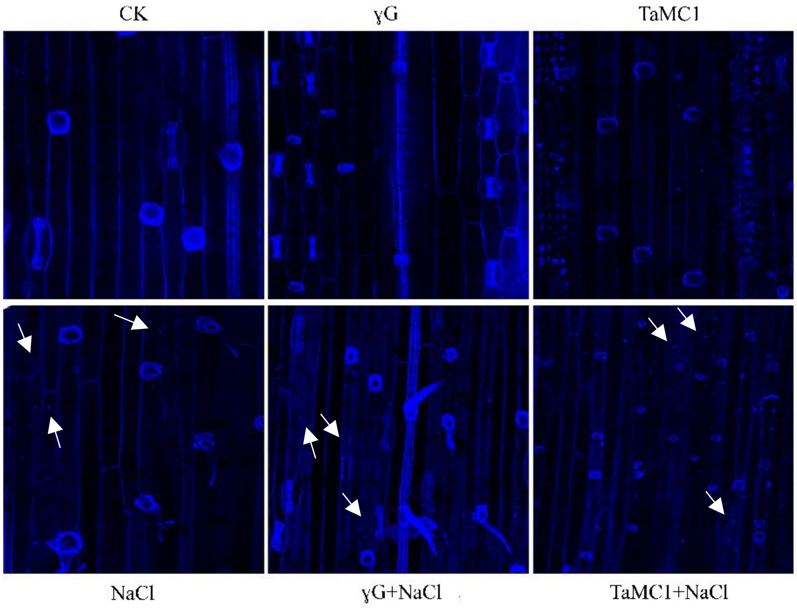

Supplementary Figure 5 The effect of silencing *TaMCA-Id* on the autophagosomes formation in leaves of wheat seedlings under NaCl stress which were stained with monodansylcadaverine (MDC). The arrows indicated the fluorescence which show the formation of autophagosomes. CK was the wild-type wheat seedlings. γG was the *BSMV-VIGS-GFP*- inoculated wheat seedlings. TaMC1 was the *BSMV-VIGS-TaMCA-Id*- inoculated wheat seedlings.

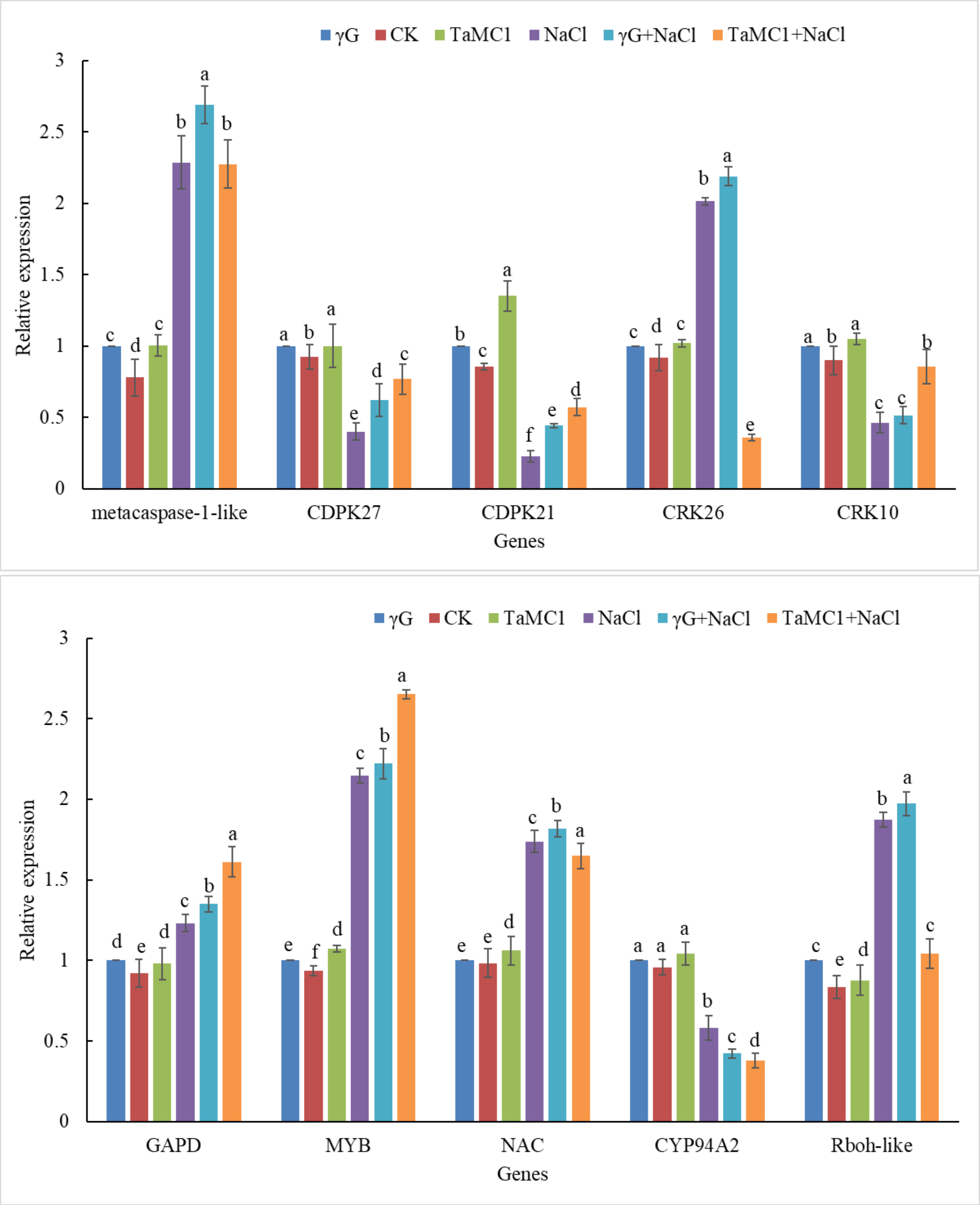

Supplementary Figure 6 The effect of silencing of *TaMC1* on the relative expression analysis of cell death- and defense-related genes in leaves of wheat seedlings under NaCl stress. Data are shown as

mean ± SD (n = 3) of three independent experiments. Bars with different letters are significantly different at $P < 0.05$. CK was the wild-type wheat seedlings. γG was the *BSMV-VIGS-GFP*-inoculated wheat seedlings. TaMC1 was the *BSMV-VIGS-TaMCA-Id*- inoculated wheat seedlings.

Supplementary Table 1 Primers used in this paper

Term	Gene	Primer sequences (forward/reverse primer)
qRT-PCR	TaMCA-Id	F: GAACGTGACGGCATGGAC<br>R: TGGTATGGGAGATCGAGGAC
qRT-PCR	ATG2	F: TGTATCCAGATGGGGGTGTT<br>R: GGAACTTAAGCTGCCCTTGA
qRT-PCR	ATG5	F: CCAGAAAGGCCATGGAATCTAAC<br>R: GCCTCTTTCAGGGAATTGTTGTA
qRT-PCR	ATG7	F: TGACGTTATCGCTCCTGTTG<br>R: ACAGCTGCTCGAGGAATAGC
qRT-PCR	ATG10	F: TATTACTCGAGAGGAGCATCCCCAC<br>R: GATTTTCAATCCTACTGCCTGACCG
qRT-PCR	NAC	F: TCTCCTCGCCACGGTTTC<br>R: GGCTGGGATTTGTTACGG
qRT-PCR	MYB	F: GACGACCCGGCGGTGAAACA<br>R: TGCATGGGCACGGAGGAAGC
qRT-PCR	ATG8	F: GGAAAGGAGGCAAGCTGAA<br>R: GCATCTCGTTAGGGACAAGGTA
qRT-PCR	glyceraldehyde-3-phosphate dehydrogenase B (GAPD)	F:TTGACCCTTCTTCTGTGAGTTG<br>R:AAAGTTGCCATCAGGAGTAAGC
qRT-PCR	cytochrome P450 94A2-like (CYP94A2)	F:AGTGCAGCTCAGGATGAGTGA<br>R:GACTAAGGACAAACCCAGAAGG
qRT-PCR	respiratory burst oxidase homolog protein B-like (Rboh-like)	F:CACCCGTTCTCCATCACA<br>R:TCTACCTGGCGAATCTCGT
qRT-PCR	metacaspase-1-like	F: AGTATGGGTGCTTGAGGTGG

		R: TGGGTCTTTCCGTTGCTG
qRT-PCR	Calcium-dependent protein kinase 27 (CDPK27)	F:GCCGCCTTCCAATACTTT<br>R:TTATCCTGATCTACTTCGCCTA
qRT-PCR	Calcium-dependent protein kinase 21 (CDPK21)	F:TTCCTGTTCGCCAACAAATC<br>R:CCTGCCCGTAGTTACGCTTC
qRT-PCR	cysteine-rich receptor-like protein kinase 26 (CRK26)	F:ATGATGATGGCCTTCTGCT<br>R:TGATGTTGCTCGGCTTGA
qRT-PCR	cysteine-rich receptor-like protein kinase 10 (CRK10)	F:GTGGCTCCATTATCGTCA<br>R:TCTTCTGGCCTATTCGTG
BMSV-VIGS	TaMCA-Id	F:CAAACATTTTTTTTTTTTTTAGCTAGCGAACGTGACGGCATGGAC<br>R: GATTCTTCTTCCGTTGCTAGCTGGTATGGGAGATCGAGGAC
Subcellular localization	TaMCA-Id	F:AAGTCCGGAGCTAGCTCTAGAATGAACTGCGGAAGCGGTCC<br>R:GCCCTTGCTCACCATGGATCCCTACATGCAGAACGGCTCGC

Supplementary Table 2 The root length and the third leaf length in *BSMV-VIGS-GFP*- (γG), *BSMV-VIGS- TaMCA-Id* - (TaMC1), and the wild-type wheat seedlings (WT) under NaCl stress

Treatments	Root length	Leaf length
WT	31.17±0.42[a]	26.70±0.33[a]
γG	30.23±0.25[b]	26.23±0.30[a]
TaMC1	29.57±0.36[c]	25.12±0.39[b]
WT+NaCl	23.18±0.44[d]	19.88±0.26[c]
γG+NaCl	22.93±0.11[d]	19.22±0.31[c]
TaMC1+NaCl	21.10±0.32[e]	18.07±0.27[d]

Note: The data are shown as mean ± SD (n = 3) of three independent experiments. The data with different letters in same column show significant difference (P <0.05).
